# Quantitative Investigation of Quantum Emitter Yield in Drop-Casted Hexagonal Boron Nitride Nanoflakes

**DOI:** 10.1021/acsaom.4c00200

**Published:** 2024-07-02

**Authors:** Tom Kretzschmar, Sebastian Ritter, Anand Kumar, Tobias Vogl, Falk Eilenberger, Falko Schmidt

**Affiliations:** † Institute of Applied Physics, Abbe Center of Photonics, Friedrich-Schiller-University, D-07745 Jena, Germany; ‡ Department of Computer Engineering, School of Computation, Information and Technology, Technical University Munich, D-80333 Munich, Germany; ¶ Munich Center for Quantum Science and Technology (MCQST), D-80799 Munich, Germany; § Fraunhofer Institute for Applied Optics and Precision Engineering IOF, D-07745 Jena, Germany; ∥ Max Planck School of Photonics, D-07745 Jena, Germany

**Keywords:** Single-Photon Emitters, Hexagonal Boron Nitride, Drop-Casting, Quantum Technologies, Fluorescent Defects

## Abstract

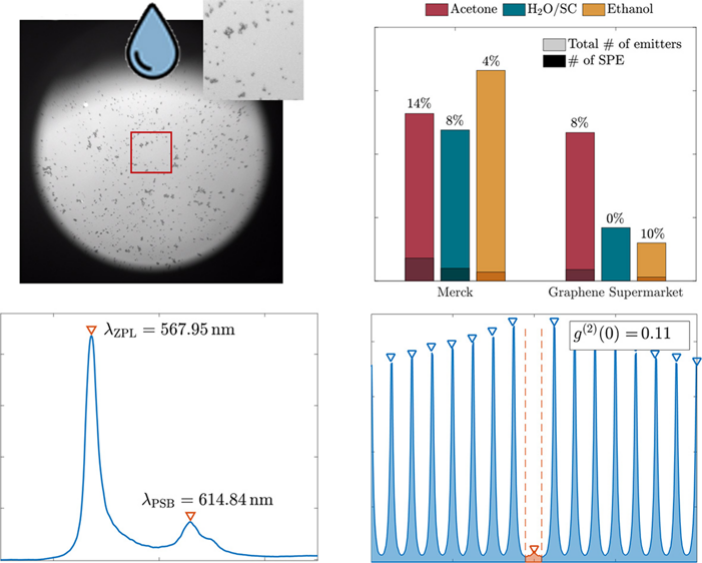

Single photon emitters (SPEs) are a key component for their use as pure photon source in quantum technologies. In this study, we investigate the generation of SPEs from drop-casted hexagonal boron nitride (hBN) nanoflakes, examining the influence of the immersion solution and the source of hBN. We show that, depending on the utilized supplier and solution, the number and quality of the emitters change. We perform a comprehensive optical characterization of the deposited nanoflakes to assess the quality of the generated SPEs. Importantly, we provide quantitative data on SPE yields, highlighting significant variations among solvents and different sources of hBN. We find that hBN from Merck drop-casted in acetone provided the best quality emitters with a *g*^(2)^ < 0.1 and photoluminescence intensities above 300 kCounts/s. Their number of SPEs among all photon emitters was also the highest, with about 14%, rendering a total yield of about 1.25% of all drop-casted flakes. These numbers hold particular significance when evaluating drop-casting as a practical method for the generation of SPEs and their deposition and incorporation within existing nanophotonic systems. By choosing appropriate solvents and source materials' quality and yield of SPEs can be significantly increased, showcasing further optimization potential for the development of future quantum applications.

## Introduction

Single-photon emitters (SPEs) have recently gained great importance for their use as pure photon sources in quantum technologies. As a result, they have become an integral component in the fields of quantum key distribution, metrology, computing, and ghost imaging.^1−5^ Two-dimensional (2D) materials have been shown to exhibit many types of defect-based SPEs. Among the potential 2D materials, hexagonal boron nitride (hBN) emerges as an excellent host material for SPEs due to its emission at room temperature, long-term stability, low cost, biocompatibility,^6^ and widespread availability.^7−10^ Current fabrication methods for SPEs from hBN typically involve manual exfoliation from pristine bulk crystals, followed by deterministic transfer for postprocessing.^11,12^ Active emission from exfoliated flakes has been demonstrated through processes such as thermal annealing,^11,12^ by stress generation on top of nanopillars,^13^ or after high-energy beam exposure of such flakes.^8,14−18^ Nevertheless, these methods are limited due to labor-intensive transfer processes. Consequently, this impedes the progress toward integrated single-photon sources in conjunction with diverse technology platforms, particularly their efficient coupling into nanophotonic systems. Several alternative methods have emerged aiming to address this challenge, including chemical vapor deposition (CVD),^13,19^ hydrothermal reaction,^20,21^ and drop-casting.^6,22−24^ These methods offer the potential for depositing flakes onto diverse substrates, irrespective of their geometric shape, and eliminate the need for precise alignment tools. While CVD methods are rapidly advancing,^25^ leading to an enhancement in the quality of synthesized crystals, drop-casting utilizes nanoflakes derived through solvent-mediated exfoliation from pristine bulk crystals. The latter bypasses the requirement for complex fabrication equipment. Drop-casting involves dispensing a droplet of a suspension of nanoflakes onto a substrate that remains on the surface after the solvent has evaporated.^26^ The choice of solvent plays a critical role, influencing the accumulation and size of suspended nanoflakes, an aspect that has only recently been investigated for hBN.^22,27−29^ Beyond the flake’s geometry, the presence of organic solvent molecules can strongly influence SPE quality by activating additional native point defects on hBN.^30^ Experimental studies utilizing drop-casting of hBN nanoflakes observed the generation of SPEs and deposited them onto various substrates and waveguides.^22,24^ Despite that these methods are capable of producing SPEs from single hBN nanoflakes, systematic approaches to assess their yield is still in its infancy.^6,31^ While previous studies investigating the influence of solvent on SPE quality and yield have utilized exfoliated flakes,^30^ similar systematic approaches for drop-casted nanoflakes are missing.^31^ Efficient SPE generation and their implementation into quantum technologies thus require further development and optimization of such processes.

This study addresses this open challenge by systematically evaluating the impact of solvent selection on the number of generated SPEs from hBN nanoflakes using the drop-casting approach. Following a comprehensive optical characterization and analysis of photoluminescence properties of the deposited nanoflakes, we have evaluated the produced SPEs based on their brightness and purity. Crucially, we report quantitative data on SPE yield and emitter quality, highlighting significant variations among solvents and different suppliers of hBN. The highest yield for SPEs, although still small in absolute quantities, has been observed for hBN nanoflakes from Merck (Sigma-Aldrich) while drop-casted in acetone solution. These specific SPEs exhibit remarkable brightness (>300 kCounts/s) alongside high purity (*g*^(2)^ < 0.15), which are comparable to SPEs derived from exfoliated flakes.^32,33^

This study presents a systematic analysis method for generating SPEs through drop-casting. It demonstrates the feasibility of identifying and enhancing bright and pure quantum sources by employing appropriately chosen solvents, providing practical yield data to better assess the efficiency of the process in quantum applications compared to other established fabrication techniques.

## Results and Discussion

In this study we investigate the generation of SPEs through drop-casting, examining the influence of both the immersion solution and the supplier of hBN. We provide a systematic workflow analysis of drop-casted hBN nanoflakes, starting with an evaluation of their spatial distribution on the substrate before conducting photoluminescence measurements using a wide-field microscope. These preliminary tests enable an initial estimation of potential SPEs without the need for time-consuming analysis of individual nanoflakes. We then perform a comprehensive characterization of the optical properties of ensembles of individual emitters using a confocal microscope, enabling a thorough assessment of the measured SPEs’ quality. Furthermore, specific yields are provided for distinct combinations of the hBN supplier and the immersion solution.

**Figure 1:**
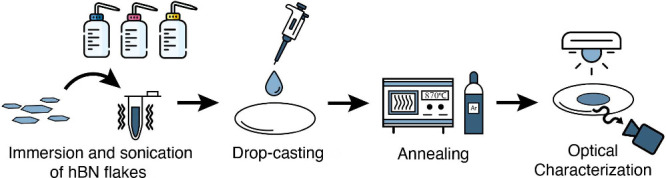
Drop-casting procedure for hBN nanoflakes. Hexagonal boron nitride (hBN) nanoflakes are first suspended in solution and shaken in an ultrasonic bath. A small droplet (*V* = 1 μL) is then drop-casted onto a substrate and dried up to 14 h overnight. The dried nanoflakes are then annealed under an argon environment in a rapid thermal annealer at 870 °C. The sample is then optically characterized using spectroscopy and brightfield and time-resolved fluorescence microscopy.

We have developed a simple recipe that reliably generates SPEs from immersed hBN nanoflakes, which is written in detail in the Methods section. In summary, nanoflakes of hBN are immersed in solution, sonicated for about 20 min, and then drop-casted onto a silicon dioxide (SiO_2_) wafer. The droplet is dried up to 14 h overnight under ambient conditions and subsequently annealed at 870 °C for 15 min under an argon environment using a rapid thermal annealer (Figure 1).

### Nanoflake Distribution

The spatial distribution of deposited nanoflakes on the substrate strongly depends on the drying process of the droplet. As larger clusters of nanoflakes may form during this process, the chances of finding those composed of only a few layers, which may potentially carry a single SPE, can be drastically reduced. To mitigate large clustering, we started by investigating the influence of the solvent on the distribution of nanoflakes.

**Figure 2:**
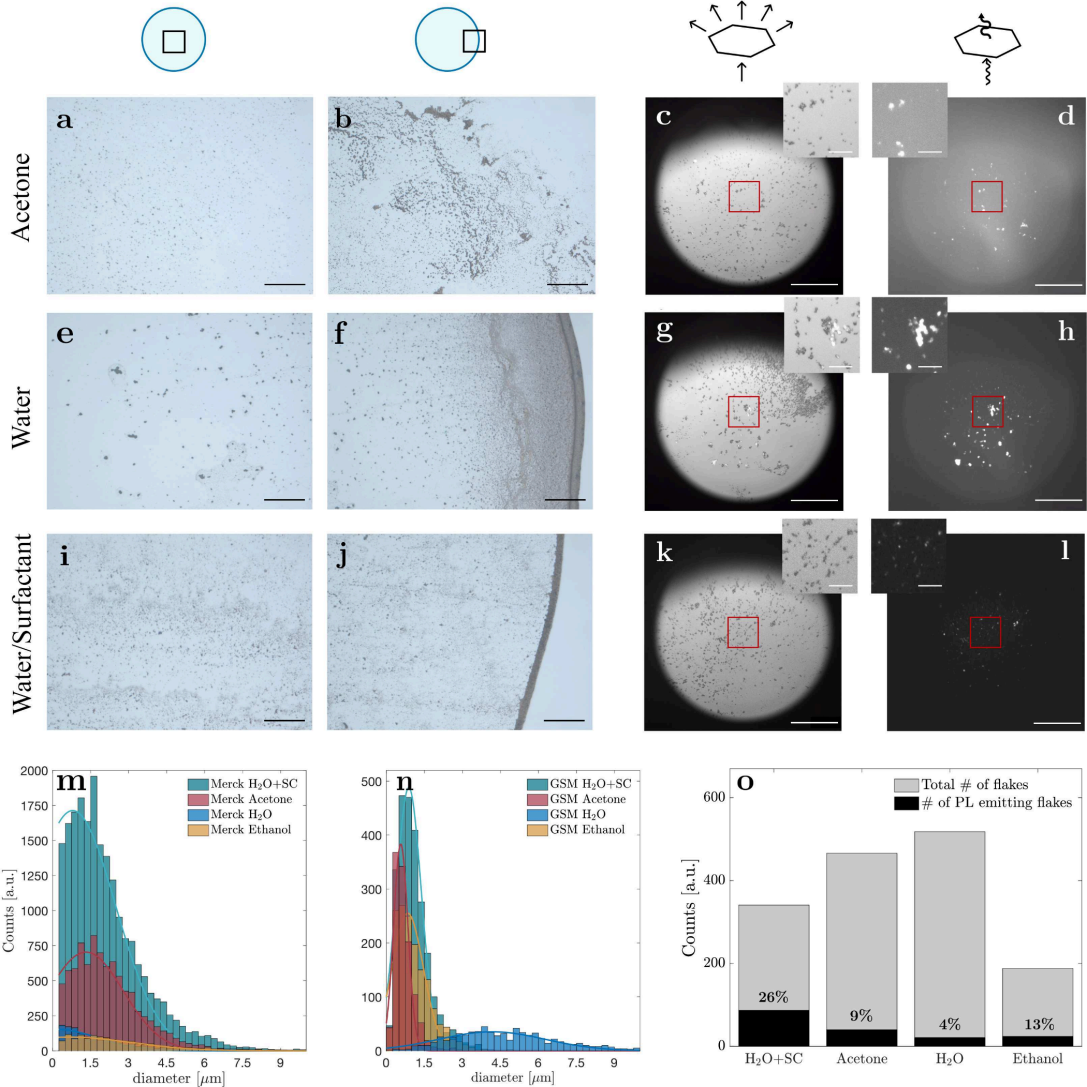
Solvent-dependence of size distribution and photoluminescence. **a**–**c** Brightfield (BF) images and **d** photoluminescence (PL) image of drop-casted nanoflakes when acetone has been used as solvent. **e**–**h** show the case when pure water was used, and **i**–**l** for water with surfactant. **a**,**e**,**i** BF images of the center of the dried droplet show a more uniform distribution of deposited nanoflakes; **b**,**f**,**j** compared to its edge, where clusters strongly accumulate. Scale bars: 100 μm. **c**,**g**,**k** Direct comparison of BF images of nanoflakes; **d**,**h**,**l** with their PL they show large differences between solvents. Scale bars: 50 μm, inset scale bars: 20 μm. **m** Size distributions of Merck’s hBN nanoflakes, **n** as well as for hBN from Graphene Supermarket (GSM), vary for different solvents with water (dark blue curve) being the broadest. **o** Ratio of PL emitting flakes (counted in **d**,**h**,**l** and SI Figure S 1d,h,l) over their total amount (counted in **c**,**g**,**k** and SI Figure S1c,g,k) for different solvents where the highest percentage was found for water and surfactant (H_2_O+SC).

During the drying of the droplet, capillary flows transport the suspended nanoflakes from the center toward the pinning line at the edge of the droplet. While some nanoflakes are deposited within the center of the droplet (Figures 2a,e,i), the majority tend to accumulate near its edge (Figures 2b,f,j). This process results in a ring-shaped distribution of nanoflakes on the substrate, known as the coffee-ring effect.^34^ Using a commercial brightfield microscope (Zeiss Axio Imager M2m) we can observe how the solution composing the droplet changes the spatial distribution of nanoflakes after drying. Our observations reveal distinct behaviors based on the immersion solvent. Specifically, when immersed in acetone (similarly for ethanol and isopropanol, detailed in SI Figure S1), nanoflakes within the central region of the dried droplet demonstrate uniform distribution and remain small (Figure 2a), which is desirable. However, the edge possesses a rather less defined structure due to a higher accumulation of clusters (Figure 2b). In stark contrast, the immersion of nanoflakes in water leads to the formation of larger clusters at the center (Figure 2e), accompanied by a pronounced manifestation of the coffee-ring effect near the droplet’s edge (Figure 2f). This result is unsurprising since hBN sheets are hydrophobic, leading to the stacking of individual layers due to van der Waals forces. Similar clustering has been observed in the case of methanol (SI Figure S1e,f). To prevent stacking, an ionic surfactant such as sodium cholate hydrate (SC) can be added to water (*c* = 47 mg/mL), where the electrostatic repulsion between surfactant molecules hinders the attachment of single hBN layers.^28^ Consequently, using water and surfactant, smaller and more evenly distributed nanoflakes are found near the droplet’s center (Figure 2i), while a ring-shaped accumulation remains at the edge (Figure 2j). The choice between hBN suppliers, whether it is Merck’s hBN powder (Figure 2 and SI Figure S1) or hBN nanoflake solution from Graphene Supermarket (SI Figure S2), both of similar size (*d* < 200 nm), does not produce visibly different spatial distributions. Other suppliers of hBN, PlasmaChem and HagenAutomation, have also been tested (see details in Discussion and Methods sections). To confirm our results, we also provide scans of our drop-casted hBN flakes using atomic force microscopy (AFM), where it becomes clear that smaller flakes with spatial extensions < 1 μm are thinner (∼10 nm) compared to small clusters (∼350 nm) (Figure S14).

For a comprehensive assessment of the impact of solvent and hBN supplier material on the size distribution within a droplet, we counted the number of nanoflakes and measured their corresponding sizes (Figures 2m,n). Among the various solvents, water with surfactant and acetone exhibited the largest number of nanoflakes per area, followed by ethanol and water for hBN suppliers from Merck (Figure 2m) and Graphene Supermarket (Figure 2n). While for Merck’s hBN the size distribution peaks below 1.5 μm for all solvents with an average width of ∼2.2 μm, hBN nanoflakes from Graphene Supermarket accumulate to small clusters of ∼0.8 μm with notably narrower distributions ∼0.7 μm. Only in the case of water is the size distribution biased toward larger values due to the accumulation of nanoflakes into large clusters (blue curves Figures 2m,n). The size distributions of isopropanol and methanol are presented in SI Figure S3. Notably, acetone and water with surfactant exhibited a large number of flakes with sizes < 1 μm, marking them as highly promising candidates for potential SPE sources.

We then use a wide-field fluorescence microscope, which allows us to quickly identify photoluminescent flakes under a broad excitation area (see Methods section). We directly compare the brightfield images (Figures 2c,g,k and SI Figures S1c,g,k) of nanoflakes near the center of the dried droplet with their corresponding photoluminescence (Figures 2d,h,l and SI Figures S1d,h,l) and quantified the number of emitters. In the case of drop-casting in acetone (Figures 2c,d), many photoluminescent nanoflakes, both in clustered formation and individually (see inset in Figures 2c,d), have been observed. While for water only large clusters exhibited emission (Figures 2g,h), the addition of surfactant revealed a large number of smaller flakes under excitation (Figures 2k,l).

By comparing the amount of nanoflakes in brightfield images (Figures 2c,g,k) with those emitting photoluminescence (Figures 2d,h,l) we find the highest percentage of photoluminescent flakes for water and surfactant (26%), followed by ethanol (13%) and acetone (9%), with only 4% for water (percentages of other solvents can be found in SI Figure S4). Although these numbers cannot differentiate SPEs and broadband emitters, they are good indicators when evaluating drop-casting methods for SPE generation. These assessments consider not only spatial distribution but also the quantity of photoluminescent nanoflakes. Utilizing these rapid identification methodologies, we determined water as an unsuitable candidate due to the formation of substantial clusters and a low count of photoluminescent emitting nanoflakes. In contrast, acetone, water with surfactant, and ethanol exhibited potential for generating a larger number of potential SPEs.

### Optical Characterization

**Figure 3:**
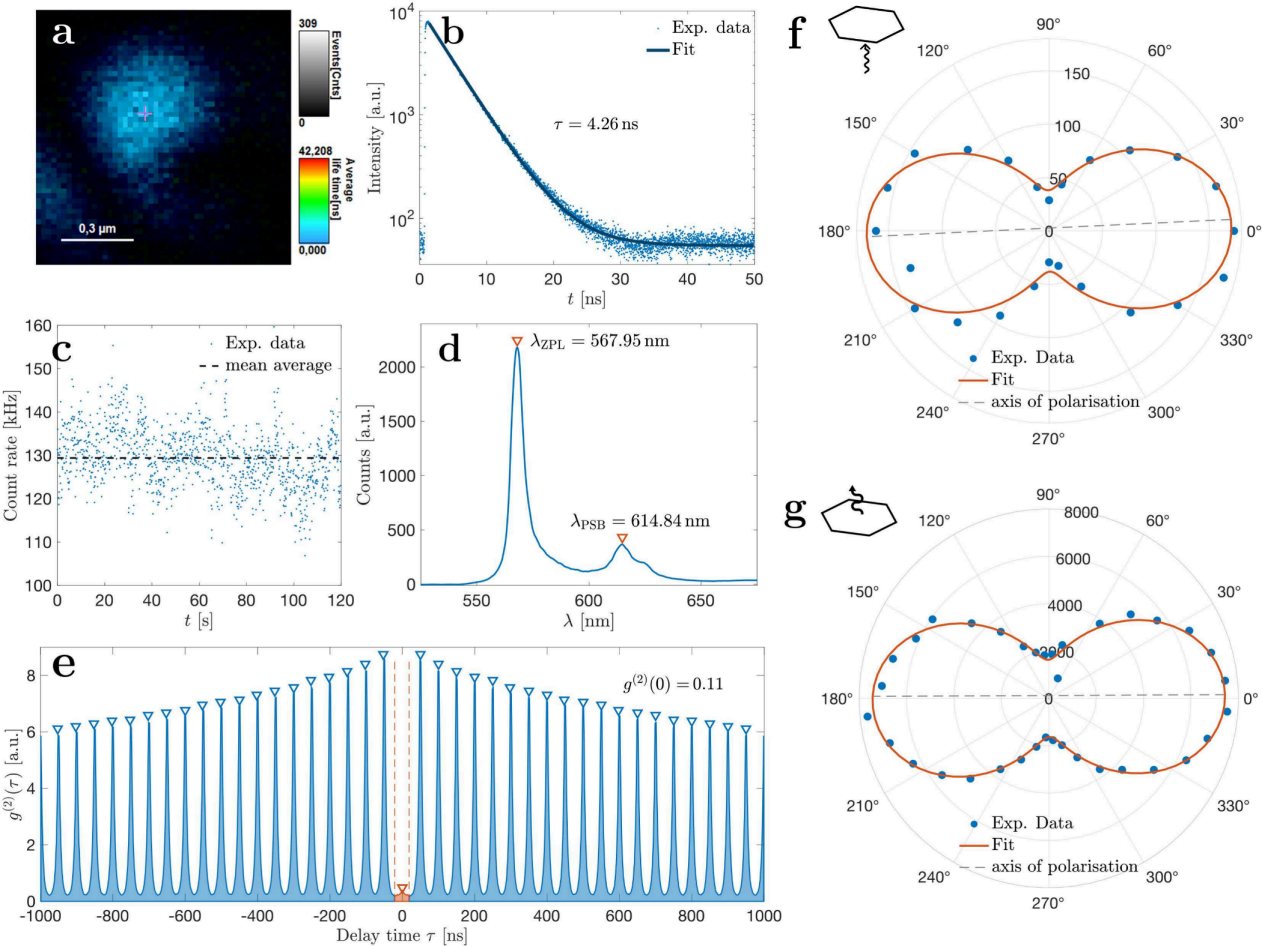
Optical characterization of Merck’s hBN drop-casted in acetone. **a** Scanned PL image of a nanoflake shows high brightness and short lifetime (blue color). **b** Lifetime analysis of the emitter reveals a decay time of τ = 4.26 ± 0.014 ns using eq 1. **c** The emitters show constant high brightness at 130 ± 8 kHz over 120 s. **d** Spectrum analysis shows a narrow zero phonon line at λ_ZPL_ = 568 nm with a smaller phonon sideband at λ_PSB_ = 615 nm. **e** Second-order time-correlated measurements *g*^(2)^ show a strong dip at τ = 0 with *g*^(2)^ = 0.11 ± 0.015, identifying these nanoflakes as SPEs. The value of *g*^(2)^(τ = 0) is calculated by taking the height of the peak at τ = 0 (orange shade) and dividing it by the average height under each other peak (blue shade). **f** Excitation polarization measurements on the PL signal, **g** as well as emission polarization measurements, reveal the dipole character of the flakes. Experimental data (blue dots) have been fitted using eq 2 (orange line), whereas the degree of linear polarization (DOP) is given by eq 3 (black dotted line). Both excitation and emission polarization axes align very well with each other.

**Figure 4:**
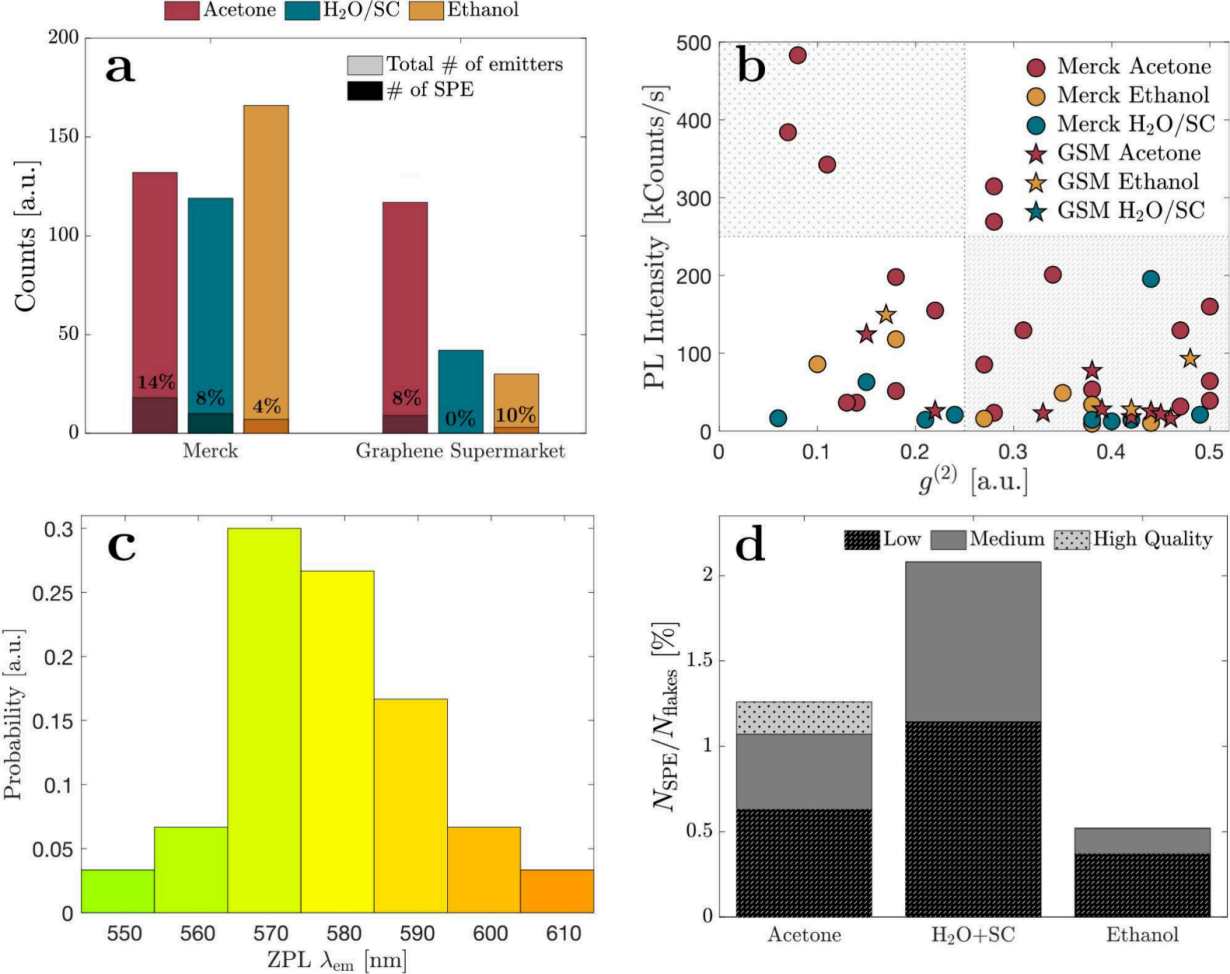
Occurence and quality of SPE. **a** Ratio of SPEs to the total number of measured emitters shows that the solvent acetone produces the highest number of SPEs in Merck, while ethanol is best for Graphene Supermarket. **b** Map of PL intensity over *g*^(2)^ spaced in four quadrants shows that acetone and Merck’s hBN combined produce the brightest and lowest *g*^(2)^ SPE. **c** SPE spectra for all combinations of solvent and hBN supplier reveal different zero phonon line (ZPL) emission between 550 and 640 nm with the highest occurrence around 570 nm. **d** Summary of the amount of SPEs identified among all deposited flakes and categorized depending on their quality. Highest quality (dotted quadrant in **b**) for emitters that are bright and possess low *g*^(2)^, medium for low intensity/low *g*^(2)^ or high intensity/high *g*^(2)^ (blank quadrants in **b**), and low quality for low intensity and high *g*^(2)^ (hatched quadrant in **b**).

We perform the optical characterization of hBN nanoflakes using a commercial confocal fluorescence microscope (PicoQuant MicroTime 200) equipped with a pair of single-photon avalanche detectors (SPAD) to measure emitter lifetime and correlations (further details in the Methods section). Employing a λ_exc_ = 530 nm laser with 10 μW power for emitter excitation, we scan the substrate using a high-NA objective (NA = 0.9), through which we can localize individual hBN nanoflakes (±5 nm) within a 80 × 80 μm area. In Figure 3 we show the results of Merck’s hBN initially immersed in acetone. For this characterization, we selected single nanoflakes with a transform-limited excitation spot (∼300 nm in size) and whose lifetime was well below 10 ns (blue color in Figure 3a). Through lifetime analysis, we find an average lifetime of around 4.26 ± 0.014 ns for this type of emitter (Figure 3b, see details in Methods), consistent with previous literature reports.^1,18^ Over an extended acquisition time of 2 min, a stable emission rate of around 130 ± 10 kHz with no signs of blinking nor bleaching was observed (Figure 3c). Spectral analysis revealed a zero phonon line at around λ_ZPL_ = 568 nm, accompanied by a phonon sideband around λ_PSB_ = 615 nm (Figure 3d). Over time, the zero phonon line (ZPL) did not change, as shown in SI Figure S12, demonstrating the strong stability of these emitters on a time scale of several minutes. Although spectral diffusion data have not been investigated here for all experimental conditions, prior systematic studies utilizing a broad range of solvents show no significant alterations to their spectra,^12,35^ which is in line with our observations at these time scales.^36^ Emission at this wavelength suggests a defect source due to carbon atoms according to the literature.^37,38^ However, slight peak position variations between measured nanoflakes (Figure 4c) indicate the potential occurrence of multiple defect types. To verify the nanoflakes as SPEs, we have performed second-order correlation measurements *g*^(2)^(τ), revealing low correlations of *g*^(2)^(τ = 0) = 0.11 ± 0.015, indicative of single-photon sources (Figure 3e). Further investigations involved examining the polarization dependence of excitation and emission of the SPEs by placing polarizers in the respective beam paths. By rotating the excitation polarization of the laser beam we find typical dipole emissions at 90° and 270° (Figure 3f) by fitting the intensities using eq 2.^38^ We find similar results for the polarization dependence of the single-photon emission (Figure 3g) and whose axis aligns with that of the excitation.

### Occurrence and Quality of SPE

Following the detailed optical characterization of a single type of emitter, i.e., Merck’s hBN in acetone, we have extended our analysis to assess ensembles of emitters resulting from various combinations of solvent with hBN sources. By measuring the correlation *g*^(2)^ at time delay zero, we discriminate between multiphoton emitters (*g*^(2)^ > 0.5) and single-photon emitters (*g*^(2)^ ≤ 0.5), thus determining the proportion of SPEs among all measured photoluminescent nanoflakes after drop-casting. For hBN from Merck, the highest yield was demonstrated with acetone, producing 18 SPEs out of 132 measured nanoflakes (14%), followed by water with surfactant with 10/132 (8%), and only 7/166 (4%) for ethanol (Figure 4a). Conversely, with hBN nanoflakes from Graphene Supermarket, slightly lower numbers were observed with only 3/30 (10%) for ethanol and 9/117 (8%) for acetone, while none were identified as SPEs for water with surfactant (Figure 4a). These results underscore the expected variations in SPE yield depending on the solvent used for drop-casting.^30^ However, our results shows that drop-casting in acetone provides better SPE yields compared to ethanol, which has typically been identified as a good candidate^31,39^ and, in fact, is often used as medium for the shipment of hBN nanoflakes. We attribute this variation to a combination of effects; first, a difference in physical characteristics such as surface tension leads to varying distributions of nanoflakes on the substrate,^27,29^ and second, the activation of defect centers is affected by the specific type of organic molecule.^30^ The latter effect has not been studied yet after the drying of the liquid and subsequent thermal annealing, which shows that further studies on defect generation using different solvents are required.

To better understand the statistical variations within the same solvent and supplier, we plotted the measured intensities against *g*^(2)^ values for all combinations (Figure 4b). By delineating the space into quadrants, the upper left quadrant, characterized by high intensities and low correlation values, is deemed most suitable for quantum applications. Notably, the combination of acetone with Merck’s hBN emerged as the sole option fulfilling both criteria, positioning it as the most suitable candidate for drop-casting SPEs (details on SPE yields and quality assessments for all measured solutions are available in SI Figure S5). Furthermore, we record multiple spectra within each solvent and supplier to identify peak emissions and determine the zero phonon line. Emissions spanning λ_em_ = 550–610 nm (Figure 4c) have been identified with the presence of multiple types of defect centers. These variations could be explained by the presence of strains^13^ or impurities within the crystal. The highest occurrence of SPE emission is found around 570 nm, indicating a defect center likely due to carbon impurities in the lattice structure of hBN, which has already been studied extensively in the literature.^37,38^ Although oxygen defects in hBN could produce central phonon line emission at similar wavelengths,^40^ it is less likely to occur given that the annealing occurred in the absence of oxygen. Interestingly, minimal variation in this range was observed among acetone, ethanol, isopropanol, and methanol or between different suppliers (SI Figure S6), implying insignificant alteration in defect centers across these variations. Finally, we show the actual yield of SPEs among deposited nanoflakes on the substrate (Figure 4d). This metric combines the count of PL emitting flakes among all deposited flakes (Figure 2o) with the percentage of SPEs (Figure 4a). We find the highest number of SPEs when drop-casted in water and surfactant (H_2_O+SC) with about 2%. However, when distinguishing between different qualities of SPEs based on the quadrants in Figure 4b, only drop-casting in acetone produced bright SPEs with a low *g*^(2)^ value at about 0.25% compared to the total number of flakes.

### Discussion on Choice of Solvent and hBN Supplier

The initial size of the suspended hBN nanoflakes is a crucial starting point. For our study, we deliberately selected nanoscopic flakes that are likely comprised of a single or few layers only. While larger hBN flakes exceeding 1 μm (such as 2D Semiconductor’s hBN powder) could theoretically be utilized, extensive ultrasonication at high powers (probe-tip immersion at 250 W) and prolonged suspension in water and surfactant failed to disperse these larger flakes and clusters efficiently (see SI Figure S7). Consequently, only a negligible amount of nanoflakes were present (*d* < 1 μm), challenging their identification due to substantially weaker photoluminescence signals compared to adjacent larger flakes and clusters. Consequently, hBN nanoflakes, whether presuspended in a solvent or in powder form, emerge as more suitable for drop-casting deposition.

Additionally, the choice of solvent significantly impacts the individual nanoflake deposition on the substrate. Varying surface tension of the droplet and solvent interaction with the hydrophobic surfaces of hBN nanoflakes can mitigate cluster formation, ensuring a more uniform deposition, particularly in the central area of the dried droplet.^27^ Optimal results were attained with water and surfactant, acetone, and ethanol, whereas pure water exhibited improper solvent behavior due to substantial clustering of flakes. Techniques like altering capillary flows or unpinning the droplet’s contact line could further alleviate the coffee-ring effect.^26^ Furthermore, the photoluminescence signal from hBN flakes significantly relies on their deposition, resulting in stronger signals from nanoflakes (e.g., water and surfactant, acetone, and ethanol) compared to larger clusters (e.g., water alone).

While size distribution and photoluminescence emission serve as good indicators, they are not comprehensive criteria for evaluating SPE creation. A full optical characterization of PL emitting nanoflakes was required to further distinguish SPEs from multiphoton emitters. The choice of solvent and hBN supplier significantly influenced SPE yield and quality, notably in intensity and purity. Besides nanoflakes from Merck and GrapheneSupermarket, two additional suppliers, HagenAutomation and PlasmaChem, were investigated without yielding a significant amount of SPE. Further material characterization of these flakes with, for example, AFM, Raman, or TEM could reveal more insights into their crystalline structure, but which is beyond the current scope of this study. Among all tested solvents and hBN suppliers, only Merck’s hBN in acetone yielded significant quantity and quality of SPEs. It is noteworthy that we exclusively considered photostable SPEs, exhibiting no bleaching or blinking during the entire measurement period (approximately 2 min).

However, only by combining both criteria, PL emission of deposited nanoflakes and SPE generation, can we accurately determine a realistic yield of SPEs through drop-casting. While our identified numbers remain low, they align well with qualitative observations from prior drop-casting experiments.^22,24^ Our stepwise analysis underscores that these low figures are not unexpected, given cluster formation during drop-casting (see Figure 2), while not all nanoflakes will carry defect centers that emit under λ_exc_ = 530 nm excitation. Moreover, as the emitters highly depend on the excitation polarization of the laser beam (Figure 3f), not all SPEs will be visible under fixed linear polarization, potentially increasing the actual count. The fundamental findings of this work, however, remain unaffected by these experimental details.

## Conclusions and Outlook

We have shown a systematic approach to study the generation of SPEs from drop-casted hBN nanoflakes demonstrating the feasibility of the methods and providing a realistic yield assessment. Our results allow for a better evaluation of current drop-casting methods compared to deterministic exfoliation and transfer methods.

The choice of solvent, alongside the supplied hBN material, notably influences SPE yield and quality. This holds particular significance for employing drop-casted nanoflakes as SPE sources in quantum communication, sensing, and imaging. The versatility of the drop-casting approach and significant reduction of involved equipment and manual labor allow for a much faster and seamless integration of SPEs into nanophotonic systems. For example, hBN drop-casting facilitates flake deposition on the tip of a single-mode fiber (SI Figures S8a–c) or on more complex structures like those of exposed core fibers (SI Figure S8d), which are difficult to access with conventional transfer techniques.

However, hurdles remain for drop-casting to become a practical solution for creating SPE sources, as the elimination of undesired emitters is essential to ensure only intended SPEs populate a given nanophotonic system. This necessitates additional steps like laser ablation^41^ or secondary transfer techniques. Nevertheless, drop-casting offers an alternative, highly controllable means of depositing hBN flakes.

## Methods

### Optical Setups

#### Time-Resolved Confocal Microscope

The optical investigation of quantum emitters is carried out using a commercial time-resolved confocal microscope (PicoQuant MicroTime 200). An excitation laser with a wavelength of 530 nm, pulse rate of 20 MHz, and excitation power of around 10 μW (measured before the objective) was used. The detection path of the setup uses various filters (long- and bandpass) to block the excitation laser with a cutoff wavelength at 550 nm. The setup is equipped with two SPADs, producing a Hanbury–Brown–Twist (HBT) interferometer. A pinhole was placed in the detection path with an aperture of 150 μm. The photoluminescence map of each nanoflake is created by scanning the sample stage with a 2 ms dwell time and a laser repetition rate of 20 MHz. The photoluminescence signal is collected using a 100× dry immersion objective with a high numerical aperture (NA) of 0.9. Through the 50:50 beamsplitter, the emission signal is split into two arms. At the end of each path, a SPAD (MDP, <50 ps time resolution, >49% detection efficiency) is placed to measure the second-order correlation function. The data analysis of the correlation function, as well as lifetime measurements, is performed with built-in software. The sample is rastered in *x*–*y* using a piezoscanner attached to a sample holder with nanometer precision. Depending on the acquisition mode (fast/slow), the spatial resolution can be increased from 20 nm down to 5 nm. The data acquisition time for these measurements was around 2 min per emitter. A spectrometer (Andor Kymera 328i-D2-sil) is attached to one of the exit ports of the optical setup to collect the emitter’s spectra.

#### Photoluminescence Wide-Field Microscope

To measure the photoluminescence of a large area, a custom-built fluorescence microscope was used (SI Figure S9). A light beam from a 530 nm laser is focused with a field lens onto the back focal plane of a 50× long working distance microscope objective (Zeiss EC Epiplan Neofluar 50×/0.55). This results in the distribution of the laser radiation on a larger area compared to the focus of a collimated beam. A long-pass dichroic mirror (Thorlabs DMLP550R) separates the signal from the excitation laser radiation, with additional filtering provided by a long-pass filter (2× Thorlabs FELH0550). The microscope images are formed onto a back-illuminated scientific CMOS camera (Teledyne Photometrics Kinetix) using a tube lens. To view the sample, a white-light illumination is also available (not shown in SI Figure S9).

### Sample Preparation

We have obtained hBN nanoflakes as a powder from Merck (790532, SigmaAldrich) with an average particle size *d* < 150 nm and as a water/ethanol solution from Graphene Supermarket with lateral sizes between 50 and 200 nm. Additionally, hBN nanoflakes in powder from HagenAutomation (*d* ∼ 70 nm), PlasmaChem (*d* ∼ 130 nm), and 2DSemiconductor (*d* ≫ 1000 nm) have been tested. In the case of hBN powder, a small quantity (<1 mg) was immersed in a 2 mL solution and subsequently shaken in an ultrasonic bath (100 W) between 20 and 30 min to prevent the formation of large clusters in solution. For hBN nanoflakes already in solution, an additional drying step was added before reimmersion in solution. We have investigated the influence of six solutions on the drop-casting of hBN: acetone, ethanol, isopropanol, and methanol (all with >99.9% purity), as well as DI water and a mixture of H_2_O with sodium cholate hydrate (*c* = 47 mg/mL) as the surfactant. A drop of 1 μL of solution is then drop-casted onto a SiO_2_ wafer and dried under ambient conditions overnight. This ensures that all remaining solution has been evaporated before annealing. The sample is then annealed following standard procedures^11^ at 870 °C for 15 min under an argon atmosphere in a rapid thermal annealer (RTA, Allwin21 Corp. AccuTherm AW610M). A heating rate of about 42 K/s was used to optimize the annealing process. Before annealing, we flush the RTA for 300 s with nitrogen gas to avoid the creation of new defects in hBN in an oxygen environment. A process overview of the temperatures and process gases can be seen in Supplementary Figure S13.

### Particle Counting

For the determination of the size distribution, the drop-casted nanoflakes have been measured via particle counting. In the brightfield images, we first chose an appropriate region of interest, typically in the center of the microscope image, before subtracting the background and applying an intensity threshold. The particles were then counted using standard algorithms after selecting only particles of interest with an effective area of 0.15–3.7 μm^2^. This ensures that no background noise nor large clusters are counted. We assumed that the measured particles were spherical in shape, which remains valid as long as their size is sufficiently small (*d* < 1 μm).

### Normalization of Second-Order Correlation Measurement

The second-order correlation *g*^(2)^ at zero delay is calculated for pulsed measurements by taking the height of the peak at τ = 0 (orange shade in Figure 3e) and dividing it by the average height of all other peaks except at τ = 0 (blue shade in Figure 3e). Note that all periods in the range of −1000 and 1000 ns time delay have been considered.

### Lifetime Analysis

The lifetime of the emitters *t* is extracted from the pulsed *g*^(2)^ measurement by fitting the experimental data with an exponential decay function:
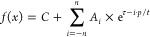
1where *C* is a constant offset in the presence of noise and *A*_*i*,*j*_ are the coefficients for growth and decay, respectively (see also SI Figure S10). Each peak is fitted due to the finite measurement time and re-emission peak dynamics.

### Polarization-Dependent Measurements

For the polarization-resolved measurements, we extended the capabilities of the commercial fluorescence lifetime microscope (PicoQuant MicroTime 200) by inserting polarizing elements in the setup, which include (i) a fixed linear polarizer in the excitation laser path to obtain horizontal polarization with a high extinction ratio, (ii) a quarter-wave plate for circularly polarized light, (iii) a polarizer that scans the emitter excitation axis after the quarter-wave plate, and (iv) a polarizer in the detection path for measuring the polarization of emission. All polarizing elements were controlled via a motorized rotation mount (Thorlabs ELL14). During these polarization measurements the laser power remained constant (SI Figure S11). The measured angular-dependent intensity of the emitter is fitted with cosine-squared function (see eq 2) to extract the axis of polarization.
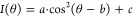
2where *a*, *b*, and *c* are the fitting parameters with θ as the axis of polarization. The degree of (linear) polarization is defined as
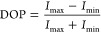
3

## Data Availability

Experimental data are available upon reasonable request.

## Supporting Information Available

Information on other combinations of solvents and hBN suppliers, as well as more details on the experimental setup and methods for rapid thermal annealing; AFM images of drop-casted flakes (PDF)
