# Supplementary material for: Quantitative Investigation of Quantum Emitter Yield in Drop-Casted Hexagonal Boron Nitride Nanoflakes
Source: ACS Appl Opt Mater. 2024 Jul 2;2(7):1427–35. doi: 10.1021/acsaom.4c00200 (PMC11287792; doi:10.1021/acsaom.4c00200)
Supplement: Supplementary file 1 — ot4c00200_si_001.pdf [file ot4c00200_si_001.pdf]

Supporting Information: Quantitative investigation of quantum emitter yield in drop-casted hexagonal boron nitride nanoflakes

Tom Kretzschmar,[†] Sebastian Ritter,[†] Anand Kumar,[†,‡,¶] Tobias Vogl,[†,‡,¶] Falk Eilenberger,[†,§,‖] and Falko Schmidt[*,†,⊥]

†*Institute of Applied Physics, Abbe Center of Photonics, Friedrich-Schiller-University, D–07745 Jena, Germany*

‡*Department of Computer Engineering, School of Computation, Information and Technology, Technical University Munich, D–80333 Munich, Germany*

¶*Munich Center for Quantum Science and Technology (MCQST), D–80799 Munich, Germany*

§*Fraunhofer Institute for Applied Optics and Precision Engineering IOF, D–07745 Jena, Germany*

‖*Max Planck School of Photonics, D–07745 Jena, Germany*

⊥*Current address: Nanophotonics Systems Laboratory, ETH Zurich, CH–8092 Zurich, Switzerland*

E-mail: schmidtfa@ethz.ch

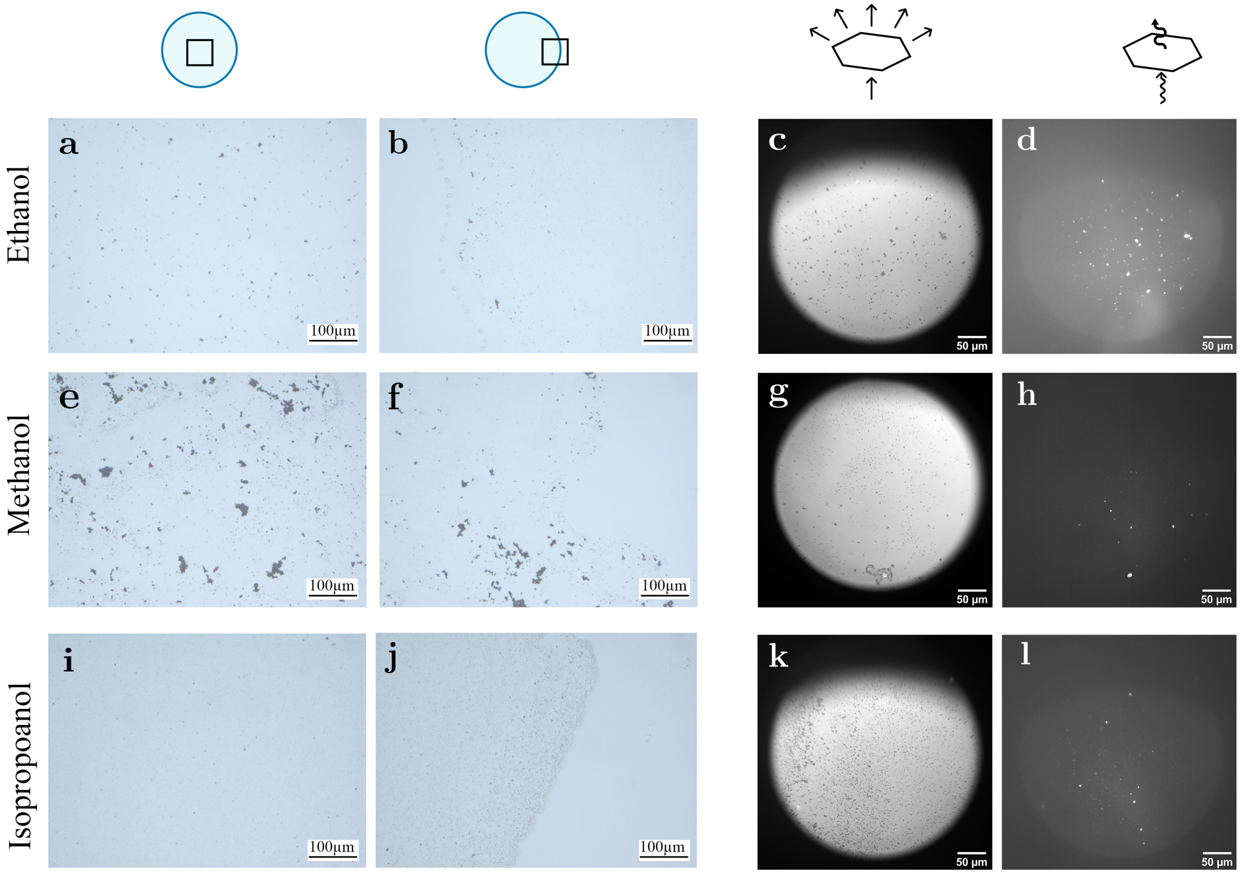

Figure S1: **Solvent-dependence of size distribution and photoluminescence for ethanol a-d, methanol e-h, and isopropanol i-l using Merck's hBN.** While for ethanol **a** and isopropanol flakes are homogeneously distributed in the center of the dried droplet, for methanol **e** large clusters are visible. **b,f,j** Although the edge of the droplet is visible no large accumulation of flakes occur. **c,g,k** Direct comparison of BF images of flakes, **d,h,l** with their PL show **d** more emitting flakes for ethanol, **h** compared to methanol and **l** isopropanol.

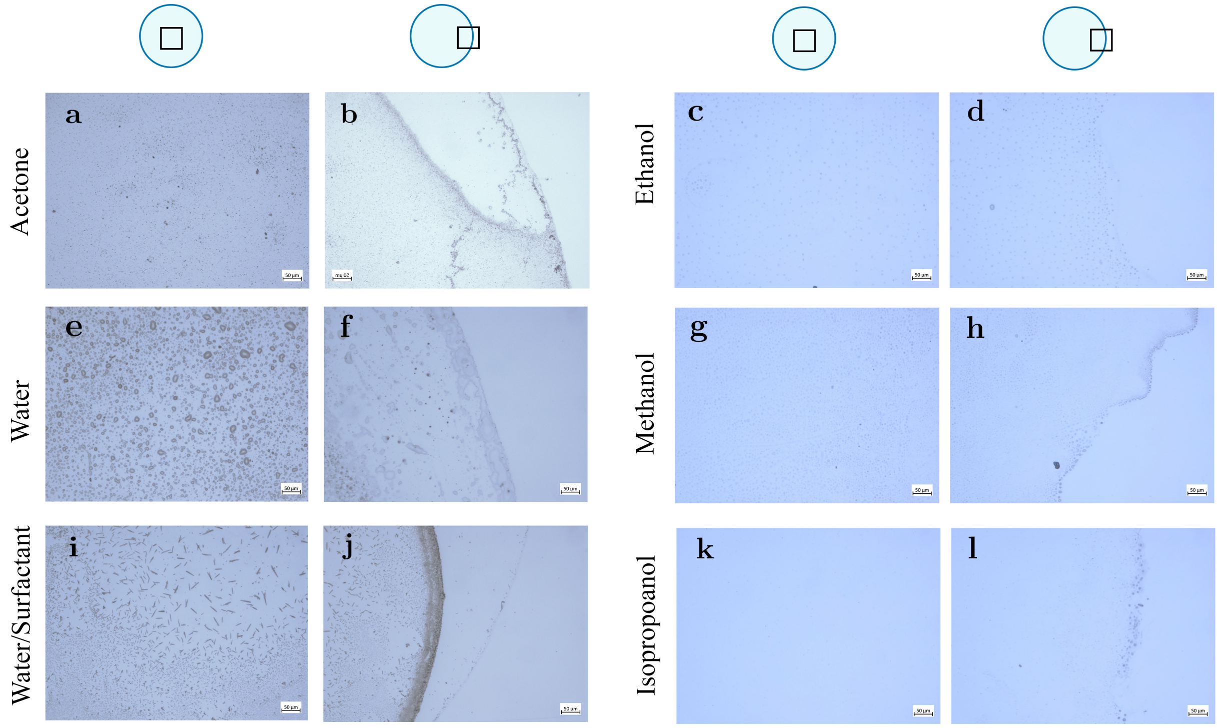

Figure S2: **Solvent-dependence of size distribution for hBN from Graphene Supermarket. a,b** For acetone smaller clusters are visible while they are **a** homogeneously distributed across the center, **b** and larger accumulations near its edge. **e** For water most clusters are formed in the center, **f** while few remain at its edge. **i** For water and surfactant particle clusters are not observed in the center but crystals form due to residue surfactant, **j** while a clear coffee-ring forms as its edge. **c,d** For ethanol, **g,h** methanol, and **k,l** isopropanol no clusters are observed in the center while smaller accumulations are found near the edge.

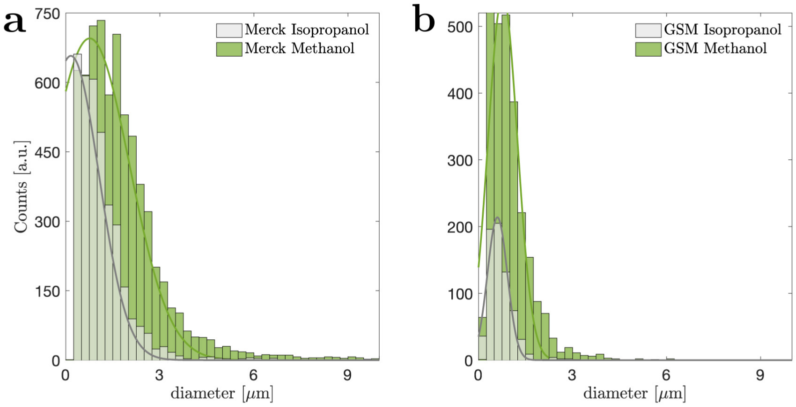

Figure S3: **Size distribution after immersion in isopropanol and methanol. a** For hBN from Merck, the size distributions of isopropanol (white) and methanol (green), and **b** for hBN from Graphene Supermarket (GSM) are all narrowly distributed below 1 μm.

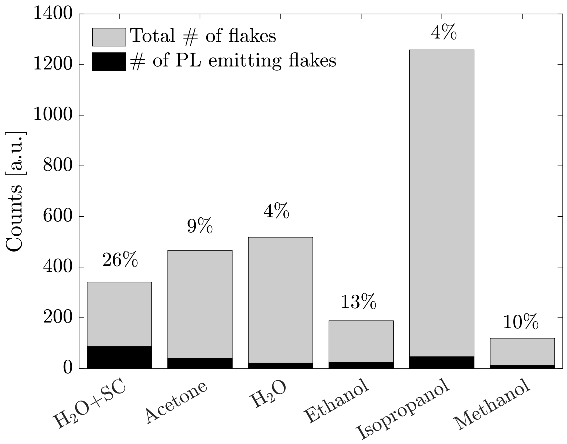

Figure S4: **Ratio of photoluminescent (PL) flakes over the total number of deposited flakes on the substrate.** Comparison of all measured solvents for Merck's hBN show highest percentages for water and surfactant (SC) whereas water (H_2O) and isopropanol have the fewest amount of PL emitting flakes.

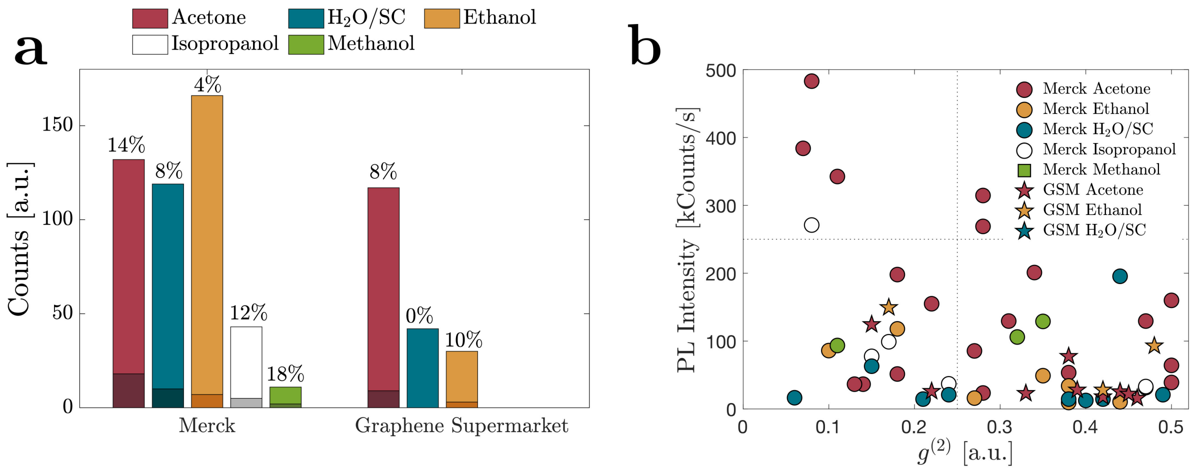

Figure S5: **SPE yield and quality characterization for all measured solvents. a** SPE ratio of all emitting flakes with additional data on isopropanol and methanol show that both possess also a high SPE ratio. **b** Measured SPE in isopropanol and methanol are mostly found among the darker emitters.

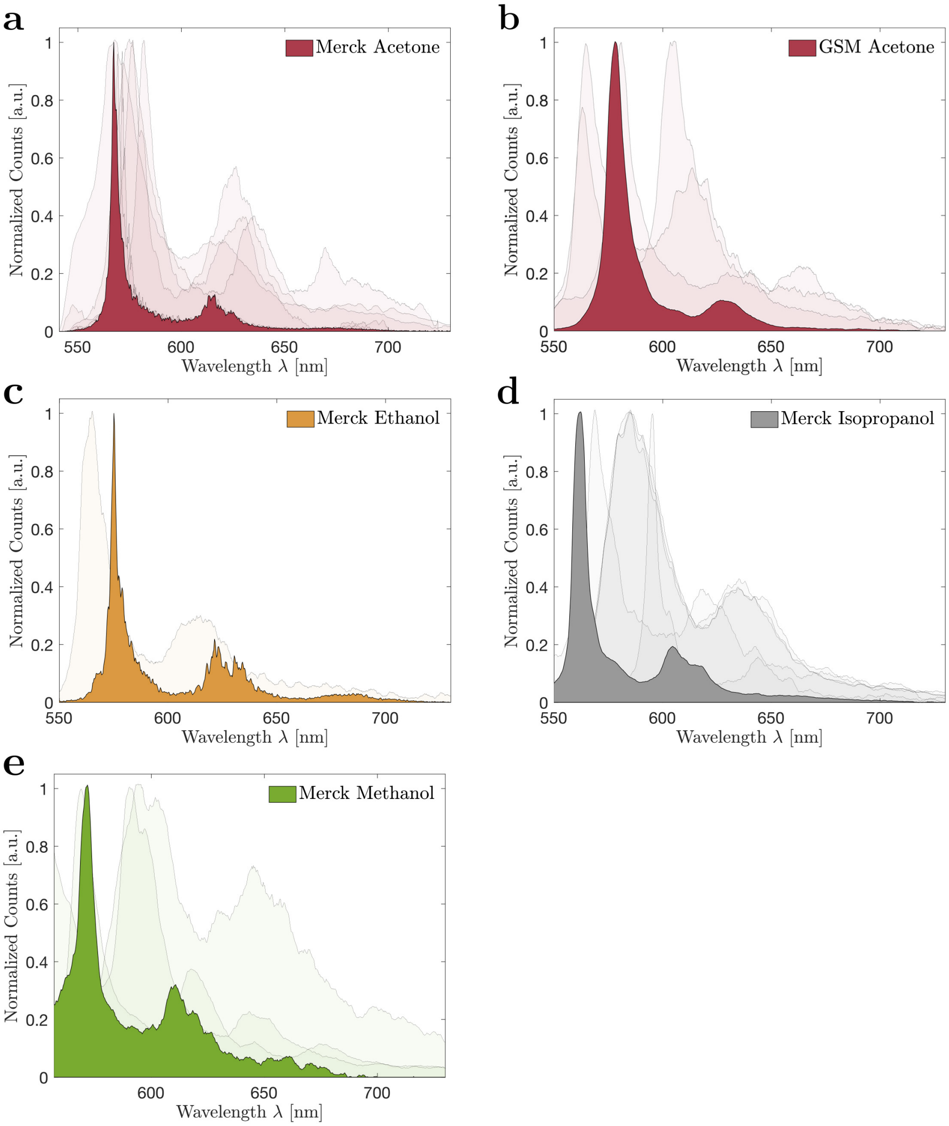

Figure S6: **Multiple recordings of spectra within each solvent a** Spectra of multiple single photon emitters in acetone for Merck's hBN, and **b** for hBN from Graphene Supermarket (GSM). Remaining spectra are for Merck's hBN in **c** ethanol, **d** isopropanol, and **e** methanol. All spectra show a similar variation of peak emissions between 550 and 600 nm.

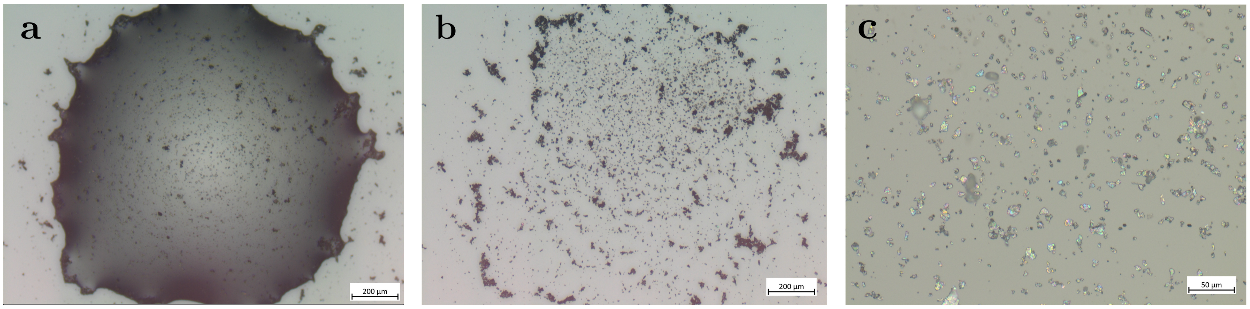

Figure S7: **Drop-casting of hBN microflakes** Using micron-sized flakes of 2D Semiconductor (PWD-HBN), **a** we take an image directly after drop-casting with droplet still visible. **b** Deposited flakes after drying of the droplet show large clusters accumulating with sizes above 10 μm. **c** Zoomed in image of the droplet in the center shows large cluster of flakes remaining on the substrate.

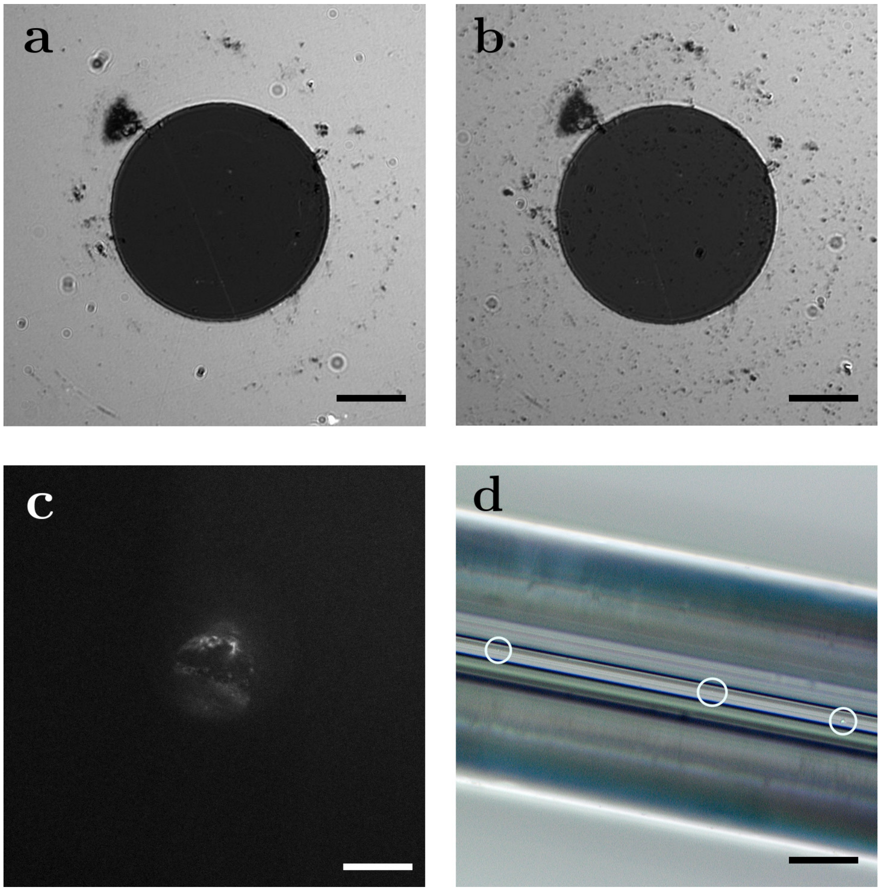

Figure S8: **Applications of drop-casting on waveguides a** The core of a single mode fiber under a 40x objective (NA = 0.7) can be **b** dipped into a suspension of hBN nanoflakes leaving a droplet on its tip. After drying, individual nanoflakes are deposited on the fiber surface (black dots). **c** Under a widefield PL microscope illumination from the other end of the fiber excites individual flakes visible as bright light source here. **d** Even unevenly shaped waveguides such as exposed core fibers can be deposited with individual flakes (white circles) directly on its core without requiring complex alignment equipment. Scalebars **a,b** 50 μm, **b,d** 100 μm.

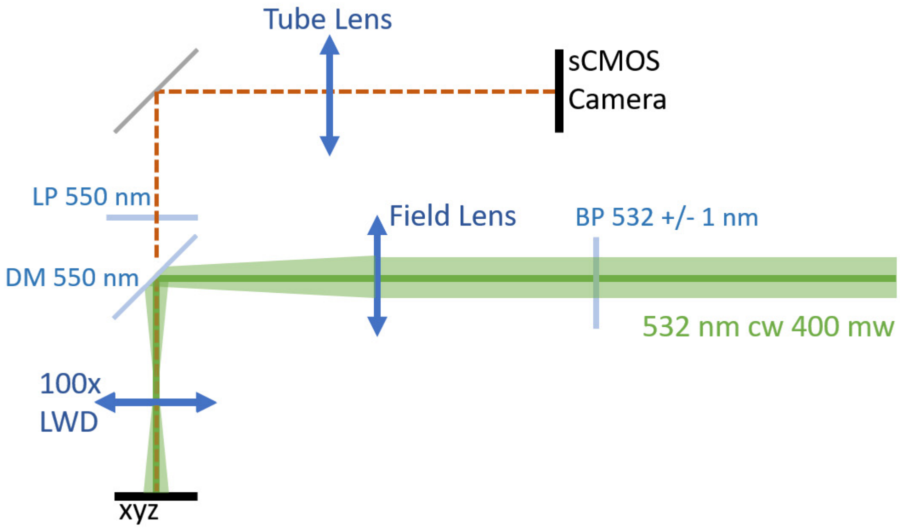

Figure S9: **Experimental setup for wide-field photoluminescence microscopy** A light beam from a CW 532 nm laser is focused with a field lens onto the back focal plane of a 100× long working distance (LWD) microscope objective. A longpass dichroic mirror (DM) and a long-pass filter only let the signal from the sample pass via a tube lens to a sCMOS camera. The sample's position can be controlled via x-y-z micrometer stage.

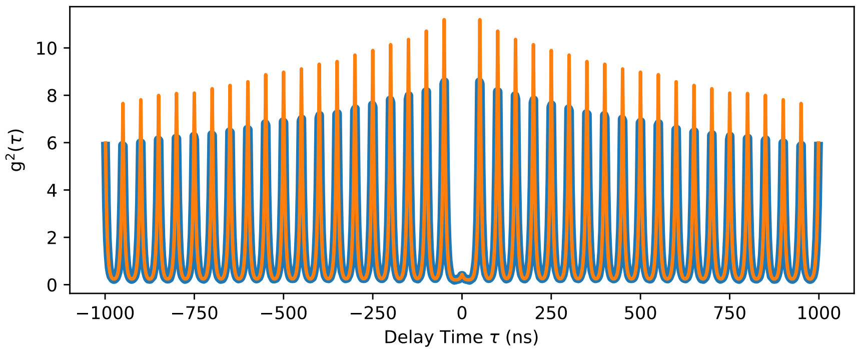

Figure S10: **Life-time analysis of SPE** Recorded $g^{(2)}$ correlation (blue line) over delay time τ for hBN from Merck and drop-casted in Acetone with fitted function (orange line) according to Eqn. 1.

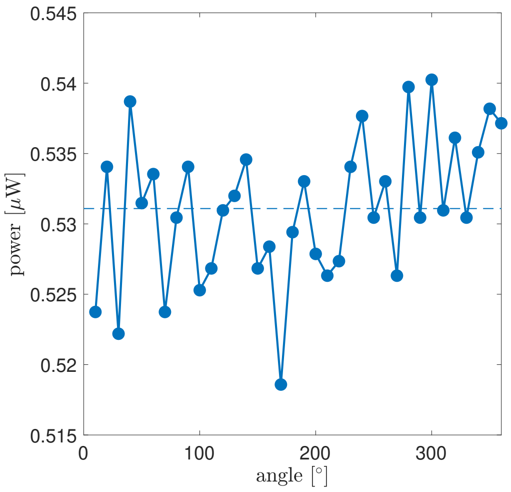

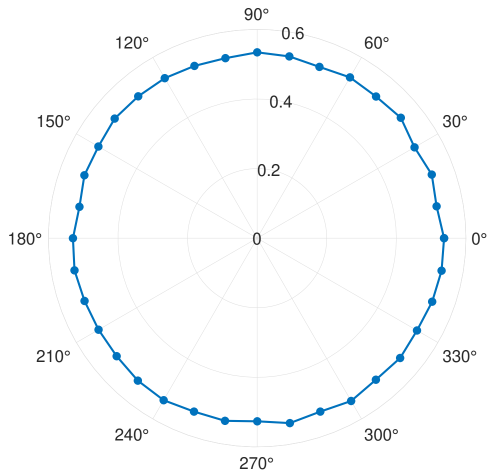

Figure S11: **Polarization dependence of laser power** The laser power remained constant over all polarization angles shown in a cartesian (left) and polar plot (right).

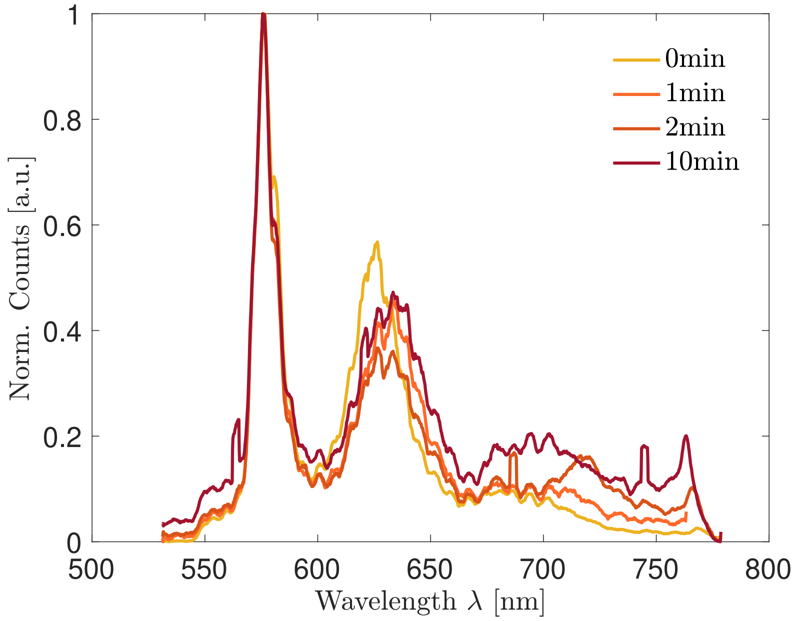

Figure S12: **Spectral change with time** Spectral change with time for a SPE from Merck and drop-casted in acetone showing no change in the position of the central phonon line with a standard deviation of the ZPL of only 0.47nm.

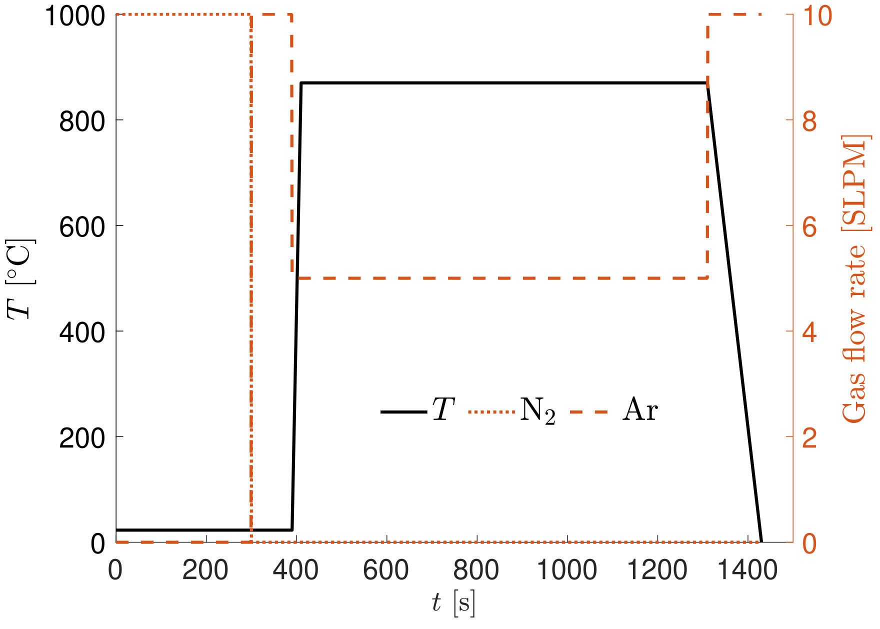

Figure S13: **Recipe for Rapid Thermal Annealer (RTA)** Recipe steps are shown including the temperature curve (black curve) as well as the flow rates of process gases (orange curves), nitrogen (N2) and argon (Ar), given in standard liter per minute (SLPM). The heating rate to the annealing temperature of 870°C was about 42 K/s.

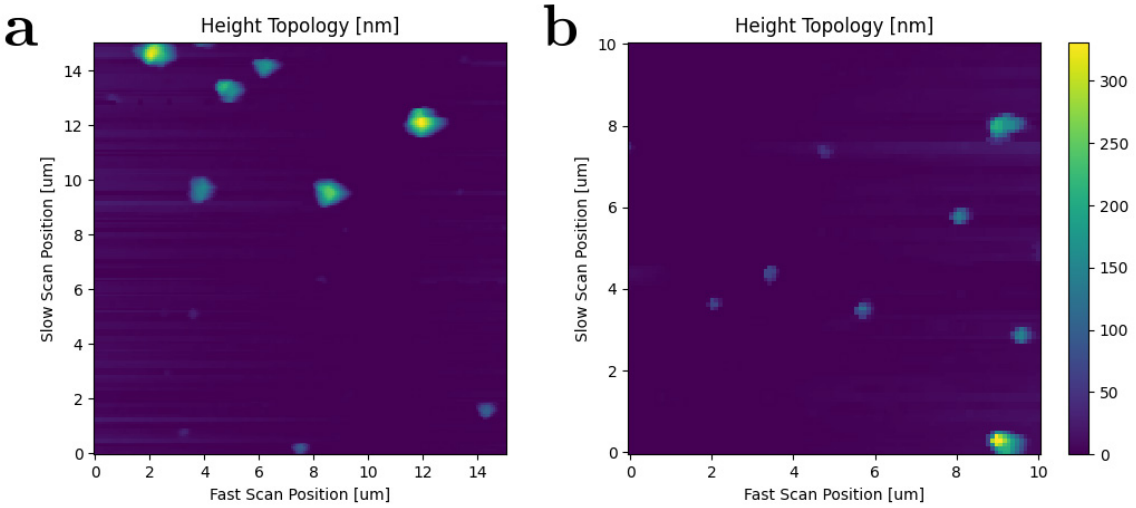

Figure S14: **Atomic Force Microscopy scans a** Small clusters and individual nanoflakes are visible when dropcasted in acetone with thicknesses ranging from 100 to 350 nm. **b** Similar types of flakes have been observed when ethanol has been used as solvent.
